# Social Skills and Reciprocal Behavior with a Virtual Player Among Children With and Without SLD/ADHD

**DOI:** 10.1007/s10578-024-01708-z

**Published:** 2024-05-15

**Authors:** Sigal Eden, Michal Ezra, Chen Rozenshtein, Sarit Alkalay, David Sarne

**Affiliations:** 1 Faculty of Education, Bar Ilan University, Ramat Gan, Israel; 2 Department of Computer Science, Bar Ilan University, Ramat Gan, Israel; 3 Department of Psychology, Max Stern Yezreel Valley Academic College, Jezreel Valley, Israel

**Keywords:** Social skills, Reciprocity, Learning disability, ADHD, Computer game

## Abstract

The study aimed to compare reciprocal behavior during interaction with a virtual-player in a computer game between children with typical development (TD) and children with specific-learning-disabilities (SLD) and/or with attention-deficit/hyperactivity disorder (ADHD), and to examine its correlation with social skills. A total of 120 children (43 SLD/ADHD, 77 TD) aged 9–11 years participated. Participants completed self-reported questionnaires focusing on social skills and reciprocity and played a computer game in which such social situations arose. Results indicated no difference between the groups in self-reported social skills or reciprocity. However, the children’s actual reciprocal behavior during gameplay revealed different results: the SLD/ADHD group exhibited higher levels of selfish (helping others for personal gain) and lower levels of altruistic reciprocity (helping others for their benefit) compared to the TD group. Furthermore, a correlation was found between self-reported social skills and reciprocity, as well as with the reciprocal-patterns observed in the gameplay.

## Introduction

The social aspects of childhood include the process of children’s identity formation and their desire to be part of an emerging peer group [4]. Children who struggle to attain these goals may suffer from reduced self-esteem and diminished self-worth, as well as difficulties in developing social connections, potentially impeding their learning processes significantly [13]. Particularly, challenges in social development are pronounced among children with specific learning disabilities (SLD) and/or attention-deficit/hyperactivity disorder (ADHD), who experience academic difficulties alongside social complexities, often have noticeable difficulties in initiating and maintaining positive relationships and effectively engaging in social interactions [47].The present study focuses on the social aspect, aiming to examine the self-reported social skills and reciprocal behavior, along with actual reciprocal behavior patterns reflected in a computer game featuring scenarios of help-providing and help-requesting. This exploration is conducted among children both with and without SLD/ADHD, with a focus on examining the correlation between these variables.

### Social Interaction Among SLD/ADHD Children

Social skills are encompass various essential dimensions such as cognition, language, and mental health, with demonstrated correlations to overall quality of life. Rooted in emotional, personal, and behavioral factors, these skills often hinge on responses to social cues and interactions, intricately tied to the social context [28, 32, 34]. Children with deficient social skills face potential adverse outcomes, including heightened vulnerability to aggression, bullying, anxiety, depression, and substance abuse, along with academic struggles and increased risk of school dropout [42]. During the primary school years, social behaviors gradually become less direct, requiring more effort in deciphering, and performing. Deeper comprehension of social and emotional nuances becomes imperative for effective engagement in interpersonal situations, necessitating executive function capabilities such as cognitive flexibility, planning, and problem-solving skills, to effectively navigate social demands within the school setting [4]. There is a prevailing consensus that cognitive understanding hinges on the ability to observe and empathize with others [32], enabling children to interpret subtle cues, take turn, adeptly transition topics in conversations, and recognize emotional expressions in others [46].

The current study focuses on children with SLD/ADHD, both high-comorbid common neurodevelopmental disorders that persist across the lifespan. Globally, approximately 5–15% of students are diagnosed with SLD with persistent learning difficulties in areas like reading, writing, arithmetic, or mathematics, while 3.5–5% are diagnosed with ADHD, characterized by sustained inattention, impulsivity, and hyperactivity [6]. Thus, both SLD and ADHD may precipitate academic underachievement, low academic performance [8, 18], as well as social and behavioral difficulties [2, 47]. Socially, these conditions can affect children’s ability to develop positive personal relationships with family, friends, peers, and romantic partners [17].

Since integrating into a peer group is a pivotal facet of identity formation, particularly during the ages of 6–12 [4], children with SLD/ADHD often encounter peer rejection, leading to fewer friendships, potentially attributed to their struggles in establishing good social skills [7, 29, 31]. Furthermore, studies indicate that some children with ADHD may exhibit aggression towards peers and authority figures, engage in disruptive behavior, property damage, conversations interruptions, heightened frustration during play, and frequent rule violations (e.g., [39]).

In recent years, there has been growing attention to defining and assessing individual differences in social abilities and interpersonal skills [33]. One aspect that promises insight into the social skills interaction of children with SLD/ADHD is reciprocity in helping behaviors. The current study endeavors to characterize the social and reciprocal behavior of this population compared to children with typical development (TD).

### Reciprocal Behavior Among SLD/ADHD

Humans are inherently prosocial beings, often engaging in behaviors that benefit both related and unrelated individuals [37]. Nevertheless, in collaborative setting, it is crucial that prosocial behaviors mutually benefit all parties involved to ensure adaptability. One way to facilitate this is reciprocal altruism [10], or reciprocity—a social preference to assist individuals conditionally based on their previous prosocial actions. This entails helping others with the expectation of future reciprocation, where the immediate costs of helping are compensated by the potential for future benefits, factored by the possibility of future collaborations [27]. Reciprocity can manifest in responding to positive actions with similarly positive ones (positive reciprocity) or responding to negative actions with corresponding negative responses (negative reciprocity) [43]. The concept of helping others is a fundamental social skill that emerges early in human development. By around 18 months of age, children begin demonstrating willingness to help, with more consistent acts of assistance observed among 3–5-years-olds, who fulfill promise of help. By age 5, children also begin to feel a sense of responsibility towards assisting others [21, 45]. Reciprocal cooperation typically develops during middle childhood, around ages 7–9 [27].

Understanding the motivations behind helping behaviors is crucial, as they vary from altruism to selfishness. Young children may act with the expectation of receiving praise or rewards when observed (selfish motives). People may help others either out of concern for themselves (egoism/selfish) or out of genuine concern for others (altruism). The interplay between the positive response of the adults watching (positive reinforcement) and children’s responses causes assimilation of helping behavior. Conversely, a child’s non-cooperation may elicit negative reactions from significant adults (parents and teachers). Thus, the presence or absence of an adult during the situation may contribute to their willingness to help [38, 45]. Helping behaviors can generally be categorized into those driven by genuine concern for other’s well-being and those guided by social norms, recognizing others’ need for help [45].

Despite the critical role of reciprocal behavior in social interactions, limited scientific attention has been devoted to studying it among children with SLD/ADHD. However, some studies have suggested that children with ADHD exhibit deficiencies in reciprocity skills and engage in less verbally reciprocal behavior [16, 19, 30]. The present study seeks to compare the reciprocal behavior patterns between the two groups—TD and SLD/ADHD children—specifically during interactions with a virtual player, with no adult’s influence. Additionally, the study explores the correlation between self-reported social skills and reciprocal behavior, as well as their actual reciprocal behavior while engaging in a computer game.

### Games, Reciprocal Behavior and Social Skills

Experiencing friendship and interaction with peers during childhood require having the appropriate skills for social play. Children’s play simulates social aspects, imparting behavioral norms and impulse control, while fostering leadership qualities [12]. Over time, various types of games have been suggested to cater to children’s cognitive and emotional needs, facilitating learning through play. These include role-playing activities [14], exercises, and other game formats [46]. Playing with a virtual player, also referred to as ‘virtual agent’, in computer games has been found to stimulate interest and enthusiasm among children. Numerous studies have shown that interacting with a virtual player provides a safe environment for children to form social connections. In particular, playing alongside a virtual player has been shown to reduce stress and anxiety levels, enabling children to learn how to create and maintain better social interactions, thereby enhancing real-life social skills [5]. Specifically, playing with a virtual player helps children with special needs comprehending social situations and enriching their repertoire of social skill [11]. Recently work of Eden and Oren [20] aimed to improve prosocial behavior among preschool children with autism spectrum disorder through computer-mediated intervention, yielding improvement in some prosocial measures. Another study involving children with various special needs, including ADHD, found that online gaming environments significantly contribute to improving positive social interactions [50].

Virtual games hold particular interest for children with ADHD as they simulate real-life scenarios. Indeed, a review by Zheng et al. [49] underscored the potential of technological games for children with ADHD, providing both enjoyment and symptom alleviation. Moreover, the coaching effect inherent in these games, contributes in transition to real-life situations. Several studies have demonstrated the efficacy of such games in enhancing social communication skills, academic performance, self-control, as well as engagement and motivation in daily activities among children with ADHD [1]. Similarly, improved learning motivation through digital games has been also found among children with SLD [24].

### The Co-Op World Computer Game

Co-Op World is an innovative game-based system designed to practice social interaction among children, focusing primarily on reciprocal-based interaction [3]. It involves virtual AI-based players utilizing advanced agent technologies, alongside a human player. Each player is tasked with collecting different types of items, coins or ice cubes, with additional score derived from collecting special items initially locked. These special items can only be unlocked by the other player, prompting the need for assistance. Providing help incurs a cost to the helping player’s score, offering no immediate reward. This scenario mirrors a wide range of real-life situations where help is required, with the helper unaware of the motives, gains, or losses of the help asker.

**Fig. 1:**
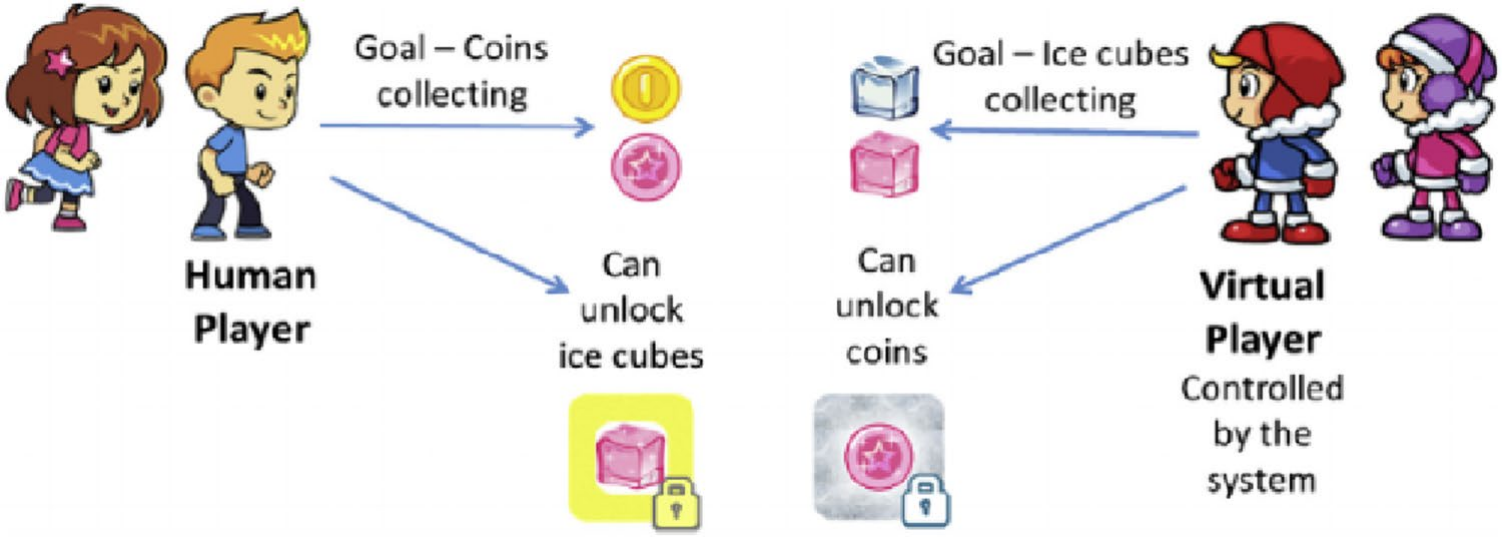
Characterizations of the Players [3]

The sole motivation for offering help lies in the hope of reciprocal assistance in future interactions with the same player. The behavior of the virtual player can be programmed in advance by the system manager, using a rich API and a predefined set of behavioral goals. This enable the integration of specific social situations throughout the game. The system’s advanced analysis tools enable comparison of results across various subset of subjects (e.g., based on age, gender, diagnosed difficulties, or any other demographic criteria), automatic clustering, anomaly detection, and visuals representations. The characterizations of the players within the game are presented in Fig. 1.

The game has successfully employed in a pilot therapy clinic [3], demonstrating its capacity to captivate the interest of primary school children and foster an understanding of reciprocity as a strategy that encourages beneficial behaviors. The examination of four case studies within that study revealed both high acceptance of the game among users and a varied set of behavioral strategies adopted by the children.

For this study, we used the game to analyze the various types of reciprocal behaviors demonstrated by the children.

### Rationale and Research Questions

Reciprocity plays a crucial role in sustaining human society and various types of social interactions [9, 23]. However, our understanding of how reciprocal behavior associates with social interaction among children with SLD/ADHD remains limited. The present study aims to advance our understanding of these issues. Several studies have indicated deficient reciprocal behavior among children with ADHD [16, 19, 30], and others have highlighted social difficulties among SLD/ADHD children (e.g., [17, 46]). Therefore, the innovative aim of this study is to examine whether patterns of reciprocal behavior differ between children with SLD/ADHD and TD children. Additionally, we seek to investigate the relationship between self-reported social skills and self-reported reciprocal behaviors, as well as those expressed within a game with a virtual player, among the two groups.

Our hypotheses were as follows:

#### H1

Children with SLD/ADHD will exhibit lower score of self-reported social skills compared to TD children.

#### H2

Children with SLD/ADHD will exhibit higher levels of selfish reciprocal behavior and lower levels of altruistic reciprocal behavior, as reported both subjectively and observed within the computer game, when compared to TD children.

#### H3

There will be a correlation between social interaction, as revealed by the self-reported questionnaire, and reciprocal behavior, as revealed by both the self-reported questionnaire and the actual computer game behaviors.

## Method

### Sample

Our sample comprised 120 elementary-school children (56 boys; 64 girls) in central Israel. Ages ranged from 9 to 12.5 years (*M* = 10.73, *SD* = 0.90). The children were sampled from two groups: (1) children diagnosed with SLD/ADHD (n = 43) and (2) TD children (n = 77). Children were divided into groups after meeting the criteria—children with TD without diagnosed difficulties based on consistent reports from the classroom teacher and parents; children with SLD/ADHD with a valid diagnosis of these disorders according to consistent reports from teachers, parents, and the diagnosis itself. All children had a normal IQ according to the diagnosis report and the school counselor’s report, and there were no additional diagnosed problems among the children, except for SLD/ADHD. Table 1 presents the children’ background characteristics.

**Table 1:** children’s background characteristics by study group

Background characteristics	Values	SLD/ADHD (n = 43)	TD (n = 77)	χ^2^	p
Gender	Boys	19 (44.2%)	37 (48.1%)	0.17	0.684
	Girls	24 (55.8%)	40 (51.9%)		
Disability	TD	0 (0.0%)	77 (100.0%)	120.00***	0.001
	SLD	9 (20.9%)	0 (0.0%)		
	ADHD	31 (72.1%)	0 (0.0%)		
	Combined SLD + ADHD	3 (7.0%)	0 (0.0%)		
School support^a^	No support	32 (74.4%)	77 (100.0%)	21.69***	0.001
	Integration teacher	6 (14.0%)	0 (0.0%)	11.31***	0.001
	Personal aide	3 (7.0%)	0 (0.0%)	5.51*	0.019
	Speech therapy	5 (11.6%)	0 (0.0%)	9.34**	0.002
	Occupational therapy	4 (9.3%)	0 (0.0%)	7.41**	0.006
	Emotional therapy	5 (11.6%)	0 (0.0%)	9.34**	0.002

^a^The percentage is over 100% since some of the children with SLD/ADHD are entitled to more than one support

*p < 0.05, **p < 0.01, ***p < 0.001

As Table 1 shows, the two study groups differ only in their disability diagnosis and school support. No significant difference was found between them regarding gender distribution. The t-test analysis for two independent samples indicated no significant difference in ages (SLD/ADHD: *M* = 10.71, *SD* = 0.83; TD: *M* = 10.75, *SD* = 0.94).

### Instruments

1. Student Social Skills Questionnaire (SSSQ, based on [26]). Administered to the children this questionnaire consisted of 34 items, with questions 9, 10, and 22 (pertaining to academic/achievement performance) removed. It divided into four domains of social behavior: (1) Collaboration, help, and communication with friends, (2) Initiating relationships, making decisions, and taking responsibility; (3) Empathy (ability to understand others’ feelings, listen, and share); (4) Ability to control emotions and behavior, reach a compromise. Responses were rated on a three-level Likert scale: *never* (0), *sometimes* (1), *often* (2). The internal consistency of all items in the questionnaire was high (α = 0.80), with each of the four scales exceeding 0.60.
2. Student Helping-Orientation Questionnaire (SHOQ, [40]). Completed by children, this questionnaire comprised 23 multiple-choice questions to determine the respondent’s propensity for one of the four helping orientation subscales: “Altruistic” (A), “Receptive-giving” (RG), “Selfish” (S), and “Inner-sustaining” (IS). Each item described a person in need and gave four possible responses reflecting the four helping behaviors. An example: *You are on the second floor of a building and notice a man stumbling around and appearing to be in trouble. Do you: (1) ignore him (IS), (2) call police fearing possible danger (S), (3) help only if you recognize him (RG), or (4) assist regardless of whether you know him (A).* Participants selected responses reflecting various helping behaviors. Each participant was scored based on the percentage of each type of response given. The internal consistency of all items in the questionnaires was high (α = 0.75).
3. Strengths and Difficulties Questionnaire (SDQ, [25]). Completed by parents, this questionnaire included 25 items across 5 sub-scales of 5 items each. Parents rated the statements on a three-level Likert scale: *not true* (1), *somewhat true* (2), *certainly true* (3). Scores for each scales ranged from 0 to 10. The internal consistency level of all items in the questionnaires was high (α = 0.83), with each of the five scales exceeding 0.70.
4. Computer Game. The study utilized the Co-Op World, a computer-based game featuring a single virtual player. Game events were automatically recorded and saved in a database for subsequent analysis. Children’s in-game behavior measures derived from the recorded data included: (1) *Help rate*—Percentage of times the child agreed to help the virtual player out of all the help requests directed from the virtual player to the child. (2) *Virtual player helped/human player helped (positive reciprocity)*—Percentage of times the child reciprocated by agreeing to help the virtual player, specifically following help requests where the virtual player helped the child. (3) *Virtual player helped/human player did not help (selfish behavior)*—Percentage of times the child refused to help the virtual player, specifically following help requests where the virtual player helped the child. (4) *Virtual player did not help/human player helped (altruistic behavior)*—Percentage of times the child agreed to help the virtual player, specifically following help requests where the virtual player refused to help the child. (5) *Virtual player did not help/human player did not help (negative reciprocity/punishment behavior)*—Percentage of times the child reciprocated by refusing to help the virtual player, specifically following help requests where the virtual player refused to help the child. Help requests were structured, with locked special elements (ice cubes or coins) appearing alternately, establishing a turn-based style for the game, where the roles of requesting help and responding to help requests are shifted between the virtual player and the human player. Collecting a special element rewards a player with a score that is ten times higher than that of collecting a regular element. Moreover, helping comes at a cost; the player who agrees to help loses five score units. The virtual player responded to help requests using a noisy tit-for-tat (TFT) strategy, typically reciprocating the human player’s last response (agreed to help/did not agree to help), but occasionally doing the opposite (with a 30% chance).

### Design

Following approval from the University Ethics Committee, the Chief Scientist of the Israel Ministry of Education, school principals, and parents, the parents were asked to complete the Strengths and Difficulties Questionnaire. Subsequently, the children, in groups of four, engaged in two 30-min sessions facilitated by the researcher to maintain their involvement. In the first session, the game was introduced, and the children participated in a trial level (1). Following this, they individually played levels 2–4 of the game, after which they completed the Helping-orientation Questionnaire. In the subsequent session, each child individually played levels 5–8 of the game and then completed the Social Skills Questionnaire*.*

## Results

Before examining the current research question, Shapiro–Wilk tests were conducted to examine whether the various research variables exhibited anormal distribution within each study group. Results indicated significant deviation from normal distribution (*p* < 0.05). Therefore, both parametric and non-parametric analyses, which do not hypothesize normal distribution of the variables, were employed to examine the research questions. Mann–Whitney tests were conducted to compare differences between the two study groups. Since the non-parametric analyses indicated the significance levels consistent with the parametric analyses, the latter’s findings are presented in this section. Mean, SD, and F-values were reported instead of the mean and sum ranks.

Before examining the research hypotheses, ANOVA analysis (on total scores) and MANOVA analysis (on subscales) were conducted to ascertain whether children diagnosed with ADHD, SLD, or a combination of both exhibited significant differences in their scores on the research variable measures. No significant differences were found across all measures [F-values ranged between 0.14 and 1.87, and p-values ranged between 0.867 and 0.167). Therefore, all further analysis will present children with SLD, children with ADHD, and children with SLD + ADHD into a single group.

### Groups Differences in Social Skills, Reciprocal Behavior, and Co-Op World Game Measures

To examine differences between the two study groups concerning social skills and self-reported reciprocal behavior (as reported by both the children and their parents), as well as reciprocal behavior measured by computer game, four one-way MANOVA analyses were conducted, one for each measure.

The results indicated significant differences between the two study groups in social skills (as reported by both children and their parents) and the children’s altruistic reciprocal behavior (helping measures in the computer game), which were all higher among the TD group [*F*(4,115) = 3.27, *p* = 0.014, *η*_*p*_^2^ = 0.10, *F*(5,114) = 10.32, *p* < 0.001, *η*_*p*_^2^ = 0.31 and *F*(2,117) = 3.68, *p* = 0.028, *η*_*p*_^2^ = 0.06, respectively]. However, no significant difference was found between groups in the self-reported reciprocal behavior [*F*(4,115) = 1.60, *p* = 0.179, *η*_*p*_^2^ = 0.05].

Additional one-way ANOVA analyses were conducted for each scale of the different study tools. Table 2 presents the mean, SD and F-values of the scores on the three questionnaires and the measures on the computer game by study group.

**Table 2:** Mean, SD, and F-values for questionnaires scores and computer game measures by study group

Variable	SLD/ADHD (n = 43)		TD (n = 77)		F	p	η_p_^2^
	M	SD	M	SD			
Self-reported social skills							
Cooperation	1.31	0.36	1.40	0.32	1.96	0.164	0.02
Initiative	1.38	0.42	1.24	0.32	4.08*	0.046	0.03
Self-control	1.13	0.35	1.15	0.33	0.15	0.701	0.00
Empathy	1.39	0.34	1.38	0.30	0.02	0.892	0.00
Total score	1.28	0.28	1.28	0.21	0.01	0.925	0.00
Strengths and difficulties (Parents)							
Emotional problems	1.60	0.50	1.33	0.36	11.07***	0.001	0.09
Conduct problems	1.38	0.36	1.20	0.28	9.25***	0.003	0.07
Hyperactivity	2.08	0.41	1.53	0.42	48.16***	0.001	0.29
Peer problems	1.42	0.49	1.29	0.35	2.67	0.105	0.02
Prosocial scale	2.62	0.42	2.70	0.33	1.55	0.216	0.01
Total score	1.57	0.29	1.33	0.21	27.07***	0.001	0.19
Self-reported reciprocal behavior							
Inner-sustaining	1.98	2.17	2.66	1.74	3.57	0.061	0.03
Selfish	1.95	1.84	2.44	1.94	1.81	0.181	0.02
Receptive-giving	4.60	1.89	5.13	2.10	1.85	0.176	0.02
Altruistic	14.44	4.21	12.75	3.98	4.76*	0.031	0.04
Total score	77.47	9.41	73.95	8.25	4.53*	0.035	0.04
Reciprocal behavior (Computer game measures) (%)							
Virtual player helped/human player helped (positive reciprocity)	66.90%	26.72	76.52%	16.74	5.87*	0.017	0.05
Virtual player helped/human player did not help (selfish behavior)	33.10%	26.72	23.48%	16.74	5.87*	0.017	0.05
Virtual player did not help/human player helped (altruistic behavior)	43.46%	25.02	51.89%	19.33	4.23*	0.042	0.04
Virtual player did not help/human player did not help (negative reciprocity/punishment behavior)	56.54%	25.02	48.11%	19.33	4.23*	0.042	0.04
Help rate	56.65%	22.62	65.85%	16.74	6.45*	0.012	0.05
						0.883	0.00

*p < 0.05, ***p < 0.001

As Table 2 indicates, children with SLD/ADHD displayed significantly more initiative and exhibited more altruistic behavior compared to the TD group according to the self-reported results. Nevertheless, their parents reported that they possess more emotional problems, conduct problems, and hyperactivity difficulties compared to the parents of TD children. However, based on parental reports, the two groups did not differ significantly in their level of peer problems and prosocial behavior. These results align with the non-significant difference between the groups in the total self-reported social skills score and the moderately significant difference in initiative.

In contrast to the self-reported reciprocal behavior results, analysis of the children’s actual reciprocal behavior in the computer game showed that, compared to TD children, those with SLD/ADHD exhibited higher selfish behavior (did not help the virtual player although being helped by the virtual player) and higher negative reciprocity/punishment behavior (did not help the virtual player after the virtual player did not help them). Conversely, children with SLD/ADHD exhibited lower altruistic behavior (helped the virtual player although the virtual player did not help) compared to TD children.

It is important to note that there was considerable variability (SDs) among both study groups in the reciprocal behavior measures obtained from the computer game, and the distribution of these measures deviated significantly from normal distribution. Despite these deviations, the non-parametric Mann–Whitney tests comparing the two study groups revealed that the differences between them remained significant.

### Correlation Between Self-Report Reciprocal Behavior and Co-Op World Game Measures

To examine the correlation between self-reported reciprocal behavior and the reciprocal behavior as measured by the computer game, Spearman correlations were conducted for the entire sample and for each group separately (see Table 3).

**Table 3:** Spearman correlations between reciprocal behavior and computer game measures

Computer game measure		Self-reported reciprocal—behavior				
		Inner sustaining	Selfish	Receptive giving	Altruistic	Total score
Help rate	All sample	−0.12	−0.09	0.03	0.08	0.11
	SLD/ADHD	−0.20	−0.20	−0.30*	0.34*	0.28
	TD	−0.16	−0.07	0.17	−0.03	0.08
Virtual player helped/Human player did not help (Selfish behavior)	All sample	0.10	0.08	0.01	−0.08	−0.09
	SLD/ADHD	0.24	0.28^#^	0.34*	−0.40**	−0.36*
	TD	0.08	−0.01	−0.15	0.09	0.01
Virtual player did not help/Human player helped (Altruistic behavior)	All sample	−0.04	−0.02	−0.03	0.02	0.04
	SLD/ADHD	0.04	0.07	−0.17	0.01	−0.03
	TD	−0.17	−0.14	−0.01	0.09	0.13

The correlation between helping behavior and percentage of “virtual player assists/participant assists” is not presented since this scale exactly complements 100% with the scale of “virtual player helped/participant did not help”

The correlation between helping behavior and the percentage of “virtual player did not help/participant did not help” is not presented since this scale complements 100% with the scale of “virtual player did not help/participant helps”

*p < 0.05; ^*#*^*p* = 0.069

As Table 3 shows, among children with SLD/ADHD, Selfish behavior in the computer game showed a positive correlation with self-reported Selfish behavior (*p* = 0.068) and a negative correlation with the Altruistic Behavior. This suggests that as Selfish reciprocal-style increases, Altruistic reciprocal-style decreases, indicating a higher percentage of instances where SLD/ADHD children did not help the virtual player, even though it helped them. Nevertheless, it should be noted that although significant correlations were found, the correlation coefficients were low to medium. Conversely, among TD children, the correlations were not significant.

### Correlation Between Self-Reported Reciprocal Behavior, Co-Op World Game Measures, and Self-Reported Social Skills

To examine the correlations between self-reported social skills and reciprocal behavior, both self-reported and measured by the computer game, Spearman correlations were calculated for the entire sample and for each group separately (see Table 4).

**Table 4:** Spearman correlations between self-reported and computer-based measures for reciprocal behavior and social skills

		Children’ self-reported social skills (Total score)	Parents self-reported strengths & difficulties (Total score)
Help rate	All sample	0.27**	−0.06
	SLD/ADHD	0.57***	0.01
	TD	0.10	0.07
Virtual player helped/Human player did not help (Selfish behavior)	All sample	−0.28**	0.03
	SLD/ADHD	−0.56***	−0.13
	TD	−0.08	−0.02
Virtual player did not help/Human player helped (Altruistic behavior)	All sample	0.15	−0.07
	SLD/ADHD	0.41**	−0.08
	TD	−0.01	0.10
Self−Reported Reciprocal Behavior			
Inner Sustaining	All sample	−0.21*	−0.08
	SLD/ADHD	−0.10	−0.20
	TD	−0.31**	0.14
Selfish	All sample	−0.08	−0.08
	SLD/ADHD	−0.15	−0.01
	TD	−0.02	−0.02
Receptive Giving	All sample	−0.22*	−0.11
	SLD/ADHD	−0.38*	0.08
	TD	−0.15	−0.09
Altruistic	All sample	0.22*	0.10
	SLD/ADHD	0.26	0.08
	TD	0.24*	−0.02
Total score	All sample	0.22*	0.09
	SLD/ADHD	0.22	0.10
	TD	0.26*	−0.08

See notes for Table 3

*p < 0.05, **p < 0.01, ***p < 0.001

As Table 4 shows, within the entire sample, a correlation was found between the self-reported social skills and both the self-reported reciprocal behaviors total score and reciprocal behaviors derived from computer data. Among children with SLD/ADHD, self-reported social skills exhibited a negative correlation with Selfish behavior and positive correlation with Altruistic reciprocal behaviors (as per computer data) and percentage of acceptance of the virtual player’s requests. These results suggest that as the social skills of children with SLD/ADHD increased, Selfish behavior decreased, while Altruistic behavior and assistance to the virtual player increased. However, no correlation was found between social skills and self-reported reciprocal behavior for this group. Conversely, among TD children, no significant correlations were found between social skills and computer game data. However, Inner-sustaining self-reported reciprocal behavior showed a negative correlation with their social skills and a positive correlation with Altruistic behavior. This implies that as the social skills of TD children increased, Inner-sustaining reciprocal behavior decreased, while Altruistic reciprocal behavior increased. Notably, in both groups, no significant correlations were found between the children’s strengths and difficulties (as reported by their parents) and their reciprocal behavior.

To examine the contribution of the children’s background characteristics, their social skills (reported by them and their parents), and their reciprocal behavior to the explained variance (EPV) of the computer game measures, two-step hierarchical regression analyses were conducted for each study group. In the first step, children’s background characteristics were entered, followed by their social skills, and their reciprocal behavior in the second step. The explanatory variables were entered into the hierarchical regression model in a stepwise manner. In this manner, only variables significantly contributed to the EPV were entered into the regression model. Table 5 presents the results of the hierarchical regression analyses.

**Table 5:** Hierarchical regression analysis for computer game measures based on children’s background characteristics, social skills, and reciprocal behavior in each study group

Explained variables	Explanatory variables	B	SE.B	β	R^2^
SLD/ADHD (n = 43)					
Help rate	Children’ social skills	48.36	10.29	0.59***	0.350***
Virtual player helped/human player did not help (selfish behavior)	Children’ social skills	−56.57	12.22	−0.59***	0.343***
Virtual player did not help/participant helped (Altruistic behavior)	Children’ social skills	35.54	12.99	0.39**	0.154**
TD (n = 77)					
% of assistance to the virtual player (total score)	–	–	–	–	–
Virtual player helped/Human player did not help (Selfish behavior)	–	–	–	–	–
Virtual player did not help/Human player helped (Altruistic behavior)	Selfish behavior	−2.33	1.12	−0.23*	0.055*

See notes for Table 3

*p < 0.05, **p < 0.01, ***p < 0.001

As Table 5 shows, the degree of social skills within the SLD/ADHD group contributed significantly to the EPV of the computer game measures. Positive β values for the Altruistic scale and percentage of overall assistance to the virtual player (help rate) indicate that children with better social skills were more tended to assist the virtual player, even in cases where it did not reciprocate. However, the negative-β coefficient for the Selfish scale suggested that children with poorer social skills tended *not* to help the virtual player after receiving help from it.

In contrast, the social skills of the TD group did not significantly affect the EPV. However, the self-reported Selfish behavior significantly contributed to 5.5% of the EPV of the Altruistic scale in the computer game. Although significant, the contribution of the self-reported Selfish behavior among TD children (15.4%), was modest compared to the contribution of the social skills among the children with SLD/ADHD (35%).

Given that the degree of social skills correlated with the computer game measures only within the SLD/ADHD group, additional hierarchical regression analyses were conducted, employing a three-step approach. In the first step, children’s background characteristics as well as the grouping variable were entered. In the second step, children’s social skills and reciprocal behavior were entered. Finally, in the third step, the interaction between the group and social skills measure was entered to examine whether the grouping variable acted as a moderator variable of the correlation between children’s social skills and computer game measures. The explanatory variables were entered into the hierarchical regression model in a stepwise manner across all three steps. Table 6 presents the results of these hierarchical regression analyses.

**Table 6:** Hierarchical regression analysis for computer game measures based on SLD/ADHD children’s background characteristics, study groups, social skills, and reciprocal behavior

Steps	Explanatory variables	B	SE.B	β	R^2^	
Help rate						
1	Study group^a^	9.20	3.62	0.23*	0.052*	0.052*
2	Study group^a^	9.31	3.45	0.23**		
	Social skills	25.72	7.14	0.31***	0.147***	0.095***
3	Study group^a^	67.11	17.90	1.66***		
	Social skills	48.36	9.72	0.58***		
	Study group × social skills	–45.07	13.71	–1.48***	0.219***	0.073***
Virtual player helped/Human player did not help (Selfish behavior)						
1	Study group^a^	–9.62	3.97	−0.21*	0.047*	0.047*
2	Study group^a^	−9.75	3.74	−0.22**		
	Social skills	−30.78	7.73	−0.34***	0.161***	0.114***
3	Study group^a^	−75.59	19.29	−1.71***		
	Social skills	−56.57	10.47	0.62***−		
	Study group × Social skills	51.34	14.78	1.54***	0.240***	0.079***
Virtual player did not help/Human player helped (Altruistic behavior)						
1	Study group^a^	8.43	4.10	0.19*	0.035*	0.035*
2	Study group^a^	8.50	4.04	0.19*		
	Social skills	17.03	8.36	0.18*	0.068*	0.033*
3	Study group^a^	55.78	21.46	1.23*		
	Social skills	35.54	11.65	0.38**		
	Study group × Social skills	−36.86	16.44	−1.08*	0.106**	0.039*

See notes for Table 3

^a^Study groups (0 = SLD/ADHD, 1 = TD)

*p < 0.05, **p < 0.01, ***p < 0.001

As Table 6 shows, consistent with the findings from the MANOVA analysis, the SLD/ADHD group exhibited a higher percentage of Selfish behavior (as indicated by the negative β coefficient) and a lower percentage of Altruistic behavior (as indicated by the positive β coefficient) compared to the TD group. Notably, the interaction between study groups and children’s social skills was significant across all reciprocal styles. These results suggest that the grouping variable acted as a moderator of the correlation between children’s social skills and their interactions with the other player in the computer game measures. In essence, the correlation between children’s social skills and their interaction within the computer game was only significant among children with SLD/ADHD and not among TD children.

## Discussion

The present study examined the differences in reciprocal behavior as expressed in social skills exhibited by children with SLD/ADHD compared to TD children, alongside investigating the correlation between reciprocal behavior and social skills in both groups, utilizing self-reported questionnaires and data obtained from a computer game.

Regarding H1 and H2, which posited that children with SLD/ADHD would exhibit lower social skills than TD children and higher Selfish reciprocal behavior alongside lower Altruistic reciprocal behavior compared to TD children, the results varied depending on the assessment method. While self-reported data indicated no significant difference between the two study groups in total social skills and reciprocal behavior scores, children with SLD/ADHD self-rated themselves higher in initiative (values were similar between the two groups with respect to cooperation, self-control, and empathy), and exhibited more altruistic behavior. However, the computer game data revealed a different picture: the SLD/ADHD group exhibited higher selfish behavior and lower altruistic behavior compared to TD children, aligning with expectation. Examining types of reciprocal behavior further found that TD children presented higher reciprocity in both situations—they helped the virtual player regardless of whether it had help them or not (higher altruistic behavior). However, children with SLD/ADHD exhibited lower reciprocity in both situations (higher selfish and punishment behaviors). These interesting findings may shed light on their social skills difficulties, consistent with previous studies [17, 46, 47]. In particular, they tend to show aggression toward peers and authority figures, be disruptive and easily frustrated during play scenarios, and frequently break rules (e.g., [39]) leading to rejection by peers and, ultimately, fewer friends [7, 29, 31].

To effectively help others, individuals must identify the other person’s character and needs [45], a skill linked to Theory of Mind (ToM), a concept that explains an individual’s cognitive and emotional abilities to attribute intentions, desires, and beliefs to both others and the self [35]. It is a fundamental function for social interaction and related to reciprocal sharing, whereas children who are better at ToM’s false-belief tasks shared more resources with others in a reciprocal sharing condition [39, 48]. Studies have found that children with SLD/ADHD often exhibit low performance in ToM false-belief tasks and difficulty understanding the feelings, thoughts, and intentions of others [17, 22, 44], which might be another explanation to their difficulties in social skills.

The disparity between self-reported reciprocal behavior and the reciprocal computer game measures among the SLD/ADHD group could be explained by social desirability bias, where respondents tend to provide socially desirable responses [15]. It seems that self-reported measures of self-esteem and social skills may be influenced by factors such as social desirability, leading children with ADHD to potentially misjudge their own strengths and weaknesses [36, 41]. Therefore, children with SLD/ADHD may lack awareness of their difficulties in reciprocal behavior, potentially explaining the discrepancy observed only among this population. In contrast, computer games provide a safe environment for authentic behavior expression and measure actual reciprocal behavior within social interaction. This suggests that studies seeking to assess behaviors of children with SLD/ADHD may yield more accurate results by utilizing computer games or observing real-life behavior rather than relying solely on traditional self-reported measures.

The findings confirm H3, indicating a correlation between self-reported social skills and reciprocal behaviors across the entire sample. As well as reciprocal behaviors according to the computer game data. Among the SLD/ADHD group, improved social skills were associated with reduced selfish behavior, increased altruistic behavior, and increased assistance to the virtual player, even when unreciprocated. However, no such correlation was found among TD children, suggesting nuanced differences in reciprocal behavior between the groups.

Although previous studies hinted at a link between social interaction and reciprocal behavior [21, 45], none directly examined reciprocal behavior in children with SLD/ADHD. The findings underscore the importance of understanding social situations for displaying reciprocal behavior, a skill that children with SLD/ADHD may struggle with [17, 46, 47]. Importantly, the lack of correlation between social interaction and reciprocal behavior among TD children suggests that, while social abilities may be impaired among those with SLD/ADHD, there is greater potential for intervention programs to promote social skills in this population. This observation underscores the need for further research to investigate the efficacy and potential of such interventions.

### Summary

This study has several limitations. Firstly, social skills were measured using a self-reported questionnaire, potentially introducing subjective biases. Also, the current study is the first to use the computer game as a research tool, necessitating further validation through subsequent studies. Moreover, it is essential to acknowledge that employing a virtual character may not inevitably translate to real-life experiences. Lastly, although children with SLD and ADHD were combined to one group because their social and reciprocal behavior reports were similar, as well as the known comorbidity, future studies should address each population specifically.

To the best of our knowledge, this study is the first to examine reciprocal behavior as it emerges in an actual social interaction during a computer game, comparing children with SLD/ADHD to those with TD. Interestingly, we observed no difference between the study groups regarding self-reported social skills or reciprocal behavior. However, actual reciprocal behavior in the game revealed that SLD/ADHD children displayed higher selfish and lower altruistic reciprocal behaviors compared to their TD counterparts. Also, a significant correlation was found between self-reported social skills and reciprocal behavior within the entire sample and specifically among children with SLD/ADHD. Further studies are needed to ascertain this correlation and determine the underlying reasons for the observed difference between the SLD/ADHD and TD groups.

## Data Availability

The data used in the research cannot be publicly shared but are available upon request.
